# Characterization of a universal screening approach for congenital CMV infection based on a highly-sensitive, quantitative, multiplex real-time PCR assay

**DOI:** 10.1371/journal.pone.0227143

**Published:** 2020-01-09

**Authors:** Angela Nagel, Emmanouela Dimitrakopoulou, Norbert Teig, Peter Kern, Thomas Lücke, Dariusz Michna, Klaus Korn, Philipp Steininger, Khalid Shahada, Katrin Neumann, Klaus Überla

**Affiliations:** 1 Institute of Clinical and Molecular Virology, University Hospital Erlangen, Erlangen, Germany; 2 Department of Otorhinolaryngology, Head and Neck Surgery, Division of Phoniatrics and Pediatric Audiology, St. Elisabeth-Hospital, Ruhr University Bochum, Bochum, Germany; 3 Department of Pediatrics, St. Josef-Hospital, Ruhr University Bochum, Bochum, Germany; 4 Department of Gynecology, St. Elisabeth-Hospital, Ruhr University Bochum, Bochum, Germany; 5 Department of Pediatrics, Elisabeth-Hospital Essen, Germany; 6 Audiology and Balance Center, Hamad Medical Corporation, Doha, Qatar; University of St Andrews, UNITED KINGDOM

## Abstract

The majority of congenital cytomegalovirus (cCMV) infections are asymptomatic at birth and therefore not diagnosed. Approximately 10–15% of these infants develop late-onset hearing loss and other developmental disorders. Implementation of a universal screening approach at birth may allow early initiation of symptomatic interventions due to a closer follow-up of infants at risk and offers the opportunity to consider treatment of late-onset disease. Real-time PCR assays for the detection of CMV DNA in buccal swab samples demonstrated feasibility and good clinical sensitivity in comparison to a rapid culture screening assay. Because most cCMV infections remain asymptomatic, a universal screening assay that stratifies CMV infected infants according to low and high risk of late-onset cCMV disease could limit the parental anxiety and reduce follow-up costs. We therefore developed and characterized a screening algorithm based on a highly-sensitive quantitative real-time PCR assay that is compatible with centralized testing of samples from universal screening and allows to determine CMV DNA load of saliva samples either as International Units (IU)/ml saliva or IU/10^5^ cell equivalents. 18 of 34 saliva samples of newborns that tested positively by the screening algorithm were confirmed by detection of CMV DNA in blood and/or urine samples obtained during the first weeks of life. All screening samples that could not be confirmed had viral loads of <2.3x10^5^ IU/ml saliva (median: 6.8x10^3^) or 1.3x10^5^ IU/10^5^ cell equivalents (median: 4.0x10^2^). The viral load of screening samples with confirmed cCMV infection ranged from 7.5x10^2^ to 8.2x10^9^ IU/ml saliva (median: 9.3x10^7^) or 1.5x10^2^ to 5.6x10^10^ IU/10^5^ cell equivalents (median: 3.5x10^6^). Clinical follow-up of these newborns with confirmed cCMV infection should reveal whether the risk of late-onset cCMV disease correlates with CMV DNA load in early life saliva samples and whether a cut-off can be defined identifying cCMV infected infants with or without risk for late-onset cCMV disease.

## Introduction

Early identification of connatal cytomegalovirus (cCMV) infections is key to prevent or mitigate the sequelae of symptomatic or asymptomatic cCMV infections by antiviral therapy, intensified monitoring and/or early initiation of supportive therapy. Two strategies have been proposed for early diagnosis of cCMV infection in newborns. The targeted screening of congenital CMV infection provides screening for newborns with a history of maternal primary infection during pregnancy, a failed hearing screening, or other symptoms suggestive of cCMV infection [1–4]. Obviously, this strategy would not identify a substantial percentage of newborns with asymptomatic cCMV infection approximately 10% of which are at risk for late-onset cCMV disease. While these cases could be identified by screening of all newborns for cCMV infection, 90% of the asymptomatic cCMV infections identified by such a universal screening would not result in late-onset cCMV disease, but would lead to years of intensified monitoring and severe parental anxiety of the affected children. These consequences of a universal screening could be limited by stratifying newborns with asymptomatic cCMV infection in those with low and high risk for late-onset cCMV disease.

Polymerase chain reaction (PCR) analyses of saliva samples obtained by buccal swabs have been proposed as a universal screening approach for cCMV infections by different studies (e.g. [5–9]). As part of the CHIMES study, 34,989 infants have been investigated for the occurrence of cCMV infection [7]. Saliva of newborns was tested for the presence of CMV using a rapid culture screening assay of liquid-saliva samples (saliva swabs in transport medium) and/or real-time PCRs of liquid-saliva samples and dried saliva (air-dry swabs resuspended in PCR-grade water) specimens. Real-time PCR was performed by directly adding small amounts of liquid/dried saliva samples to the PCR (without prior desoxyribonucleic acid (DNA) extraction) and results were analyzed qualitatively to evaluate sensitivity and specificity of PCR for detecting CMV DNA in saliva specimens. In this study comparison of a rapid culture screening assay with this PCR-based assay of liquid-saliva samples identified 93 infants being positive for at least one of the two assays. All 85 infants positive for the rapid culture screening assay were tested positively by the PCR-based assay. Analyzing 7 of the 8 samples that were only positive by the PCR assay for the presence of CMV DNA in urine or blood indicated a false positive screening result for 6 of the samples. Similar results were observed using dried saliva swabs. Furthermore, subsequent analyses of the same group investigating in total 73,239 infants regarding CMV DNA detection in saliva revealed 284 positive screening results, with 18 of them classified as false positive [8]. Contamination of neonatal saliva samples by CMV DNA present in genital secretions in the birth canal during delivery [10] or in the milk remaining from the last breast feeding [11] as well as oral virus shedding due to self-limited local transient infection of the oral cavity of newborns after exposure to CMV containing genital fluids or breast milk without systemic primary infection [12] may explain these results. Because the amounts of contaminating CMV DNA should be rather small, a quantitative analysis of the CMV DNA amounts in the neonatal samples from the oral cavity may allow defining a threshold of viral DNA levels in saliva above which contamination by genital secretions or breast milk can be largely excluded. In addition, a quantitative analysis of CMV DNA loads may allow to assess the risk of asymptomatic cCMV infected neonates to develop late onset CMV disease. We therefore evaluated a real-time PCR assay controlling PCR inhibition and simultaneously quantifying CMV DNA and human genomic DNA from buccal swab samples of neonates.

## Material and methods

### Inclusion criteria and study design

Participants for the study, which was approved by the Ethics Committee of the Ruhr University Bochum (reference number: 5106–14), were recruited from October 2015 to December 2017 at the birth clinic and neonatal ward of the Katholisches Klinikum Bochum and the Elisabeth Krankenhaus Essen, Germany. Parents or caregivers of the 6,102 included neonates of the two participating hospitals have given consent prior to inclusion of their children into the study. Buccal swabs were generally taken within the first three days of life and mothers had been instructed not to breast feed two hours prior to sampling in order to avoid false positive test results from CMV DNA positive mother´s milk. Parents of babies screened positively for CMV DNA were invited for confirmatory diagnostics consisting of CMV DNA detection in urine, blood, and a second buccal swab. Samples for confirmatory diagnostics from asymptomatic children were obtained usually within eight weeks after birth. In a single case the time interval between birth and sampling for confirmatory diagnostics was 16 weeks. Because test results were not utilized to assess birth prevalence of cCMV but only for methodical analysis results from this participant were not excluded.

### Sample collection

#### Saliva samples

Consistent with a previous study, which evaluated the performance of different swabbing materials and transport time with regard to the efficacy of CMV DNA recovery [13], buccal swabs were obtained with the eNAT^™^ kit consisting of 1 ml eNAT^™^ transport and preservation medium in 12x80mm screw cap tubes and a regular FLOQSwab^™^ (Copan, Brescia, Italy, order number eNAT^™^ kit: 608CS01R). Swabs were immediately immersed in the eNAT^™^ medium, stored at 4°C, and shipped weekly from the screening clinics at ambient temperature to the Institute of Clinical and Molecular Virology at the University Hospital Erlangen, Germany (central study laboratory) for CMV DNA analyses. There was no evidence for the presence of inhibitors in the eNAT^™^ medium since Herpesvirus saimiri (HVS) DNA, which served as internal control, could be detected in appropriate amounts.

#### Blood and urine samples

EDTA blood (1 ml) and urine sample (1 ml, sterile container without additives) were collected for confirmatory diagnostics. With few exceptions, where blood and urine were sent immediately after sampling, material was stored at -20°C until shipment with coolpacks to the Institute of Clinical and Molecular Virology at the University Hospital Erlangen.

### Molecular diagnostics

All molecular diagnostics were performed at the diagnostic laboratory of the above named institute. The laboratory follows strict quality assurance principles and is accredited by the national accreditation body for the Federal Republic of Germany (DAkkS) for the diagnostic laboratory and the CMV DNA analysis based on international standards regarding requirements for quality and competence of medical laboratories (ISO 15189).

The DNA was extracted from 200 μl of eNAT^™^ samples, whole blood, or urine using the QIAsymphony^®^ DSP Virus/Pathogen Mini Kit, performed with a QIAsymphony automated extraction machine according to the manufacturer´s instructions (Qiagen, Hilden, Germany). Saliva samples were eluted in 60 μl, blood and urine samples in 110 μl. As positive control, CMV containing cell culture supernatant was used, distilled water served as negative control (Sigma, Taufkirchen, Germany). Prior to the DNA extraction, 4 μl cell culture supernatant containing approximately 2,000 copies of HVS DNA was added to each sample acting as control for extraction and PCR inhibition. Extracted DNA was amplified by a real-time PCR with primers specific for the immediate-early 1 (IE-1) gene of CMV (Table 1). Furthermore primers for cellular DNA (albumin) and HVS as internal controls were used. For detection of each of the amplicons dye-labelled hydrolysis probes were included (Table 1).

**Table 1:** Oligonucleotides of the CMV DNA PCR assay.

Assay	Sequence (5´-3´)	Position	GenBank^a^
**HCMV (IE-1 Gene, Exon 4)**			
5´ TaqMan Primer	GAG CAG ACT CTC AGA GGA TCG G	172321–172342	BK 000394.5
3´ TaqMan Primer	AAG CGG CCT CTG ATA ACC AAG	172439–172419	
TaqMan Probe	5´ FAM-CAT GCA GAT CTC CTC AAT GCG GCG-TAMRA 3´	172371–172394	
5´ PCR Primer^b^	TCT CAG CCA CAA TTA CTG AGG ACA GAG GGA	172151–172180	
3´ PCR Primer^b^	GGT CAC TAG TGA CGC TTG TAT GAT GAC CA	172551–172523	
**Albumin (Albumin Gene, Exon 12)**			
5´ TaqMan Primer	GTG AAC AGG CGA CCA TGC T	15621–15639	M12523.1
3´ TaqMan Primer	GCA TGG AAG GTG AAT GTT TCA G	15709–15688	
TaqMan Probe	5´ VIC-TCA GCT CTG GAA GTC GAT GAA ACA TAC GTT C-TAMRA 3´	15642–15672	
5´ PCR Primer^b^	CCA GTA AGT GAC AGA GTC AC	15579–15598	
3´ PCR Primer^b^	TGA TTT GTC TCT CCT TCT CAG	15747–15727	
**HVS (Major Capsid Protein, ORF25)**			
5´ TaqMan Primer	CTC ATT ACC AGA CCC ATG TTA TGA A	45251–45275	AJ410493.1
3´ TaqMan Primer	CCA TTT GCC TGT GTT GAG AGT TAA	45357–45334	
TaqMan Probe	5´ Cy5-CTC CGA GAG AGC CTA TCT GAG ATG CCC-BHQ-2 3´	45324–45298	

(modified according to [14]). IE-1, immediate-early 1; HVS, Herpesvirus saimiri; ORF, open reading frame.

^**a**^ GenBank accession number.

^b^ PCR primers for generation of quantitative standards.

Real-time PCR reactions were set up in a clean room with pipettes used specifically for this purpose. Master Mix preparations were made in sterile tubes containing 25 μl TaqMan^®^ Universal PCR Master Mix (Thermo Fisher Scientific, Darmstadt, Germany), 2 μl of each forward and reverse primer of CMV and HVS (5μM), 1.5 μl of each forward and reverse primer of albumin (1 μM), 1 μl CMV and albumin probe (10μM), 0.5 μl of HVS probe (10μM) and 1.5 μl distilled, deionized water (Sigma, Taufkirchen, Germany), for each reaction. Thus the total volume of this master mix was 40 μl. For amplification 10 μl of the DNA extracts were added to 40 μl of the master mix. The cycler (ABI Prism^®^ 7500, Thermo Fisher Scientific, Darmstadt, Germany) conditions were 2 min at 50°C, 10 min at 95°C followed by 40 cycles of 15 sec at 95°C and 1 min at 60°C. Quantification of CMV DNA was performed using a standard curve with known amounts of an IE-1 CMV PCR product (respective primers see Table 1) generated from a CMV positive patient sample. The International Unit (IU) of the CMV standard DNA had been calibrated utilizing the WHO International Standard (NIBSC code: 09/162), allowing to express all results as IUs. Albumin DNA was quantified applying a standard curve generated from an amplicon originating from a clinical sample (selected primers see Table 1). The lower detection limit in all assays was between 5 and 10 DNA copies per reaction, i.e. particularly for CMV the lower detection limit was 5 IU per reaction which equals 150 IU/ml for saliva and 250 IU/ml for blood and urine, respectively. CMV DNA load can be expressed as IU/PCR reaction, IU/10^5^ cell equivalents by calculation of the cell number contained in the buccal swab (detected by albumin DNA content) or IU/ml saliva, since FLOQSwabs^™^ of the eNAT^™^ kit absorb on average 40 mg (= 40 μl) saliva (own measurement). Of note, quantification of albumin and HVS DNA content in samples with high CMV load (>5x10^5^ IU/PCR reaction) was partially unreliable probably due to competition for PCR reagents (S1 Table).

All samples that were tested positively with <25 IU CMV DNA per PCR reaction (to avoid false positive test results), contained less than 50 copies of albumin per PCR reaction (cellular control to confirm potentially false negative results due to inefficient sampling), or had strongly reduced quantities of HVS DNA (internal control to avoid false negative results due to DNA extraction and PCR inhibition) were retested. The threshold for CMV DNA (25 IU/PCR reaction) was selected, because initial tests had shown, that samples containing ≥25 IU CMV DNA/PCR reaction were consistently positive after retesting. The threshold for albumin DNA (50 copies/PCR reaction) was arbitrarily set and excludes samples with albumin DNA values, which were >3 standard deviations under the mean of the albumin DNA levels. In case of retesting of the saliva samples, a second DNA extraction from 200 μl eNAT^™^ of the initial screening sample was performed using the EZ1 Virus Mini Kit v2.0 on a EZ1 Advanced XL automated extraction machine (Qiagen, Hilden, Germany) as described by the manufacturer. Nucleic acids were also eluted in 60 μl, 10 μl of which were used in the real-time PCR reaction as described above.

### Evaluation of results and statistical analysis

Considering the non-normal distribution of the generated data (assessed by the Shapiro-Wilk test), non-parametric tests and illustrations were used. Continuous variables are shown as scatter plots and median values are represented by black lines. For comparison of two independent groups of participants (CMV DNA load in buccal swabs of cCMV infected *versus* non-infected newborns at time of screening test) the two-sided Mann-Whitney-U-Test was used. Paired samples of the same material at two time points (CMV DNA load in buccal swabs at time of screening and confirmatory testing) were compared introducing the Wilcoxon signed rank test. For comparison of the CMV DNA load in different materials at time of confirmatory diagnostics the Friedman test was performed at first as an omnibus test for testing the difference between several (more than two) related samples (buccal swab *versus* EDTA blood *versus* urine). In the case of p<0.05, selective comparison of the CMV DNA load in saliva *versus* urine samples at time of confirmatory diagnostics was performed using the Wilcoxon signed rank test. The p-values for pairwise comparisons were not adjusted for multiple testing. Correlation analysis was quantified using the Spearman´s rank correlation coefficient. In all these tests level of significance α was defined as less than 0.05 (p<0.05). Presentation of results and statistical analysis were performed using GraphPad Prism^®^ 6.01 (GraphPad Software, USA).

## Results

### Test procedure of cCMV screening and confirmation

For ease of handling during sampling and transport to centralized screening facilities we used commercially available flocked swabs and immediately stabilized the recovered material by immersion of the tip of the swab in the denaturing eNAT^™^ medium present in the transport tube. The manufacturer warrants a 100% DNA stabilization at room temperature or 4°C for four weeks. In a study exploring the influence of pre-analytic factors, this approach has revealed the best CMV DNA recovery efficiency compared to a viral transport medium with or without prior DNA extraction ([13], S2 Table).

**Fig 1:**
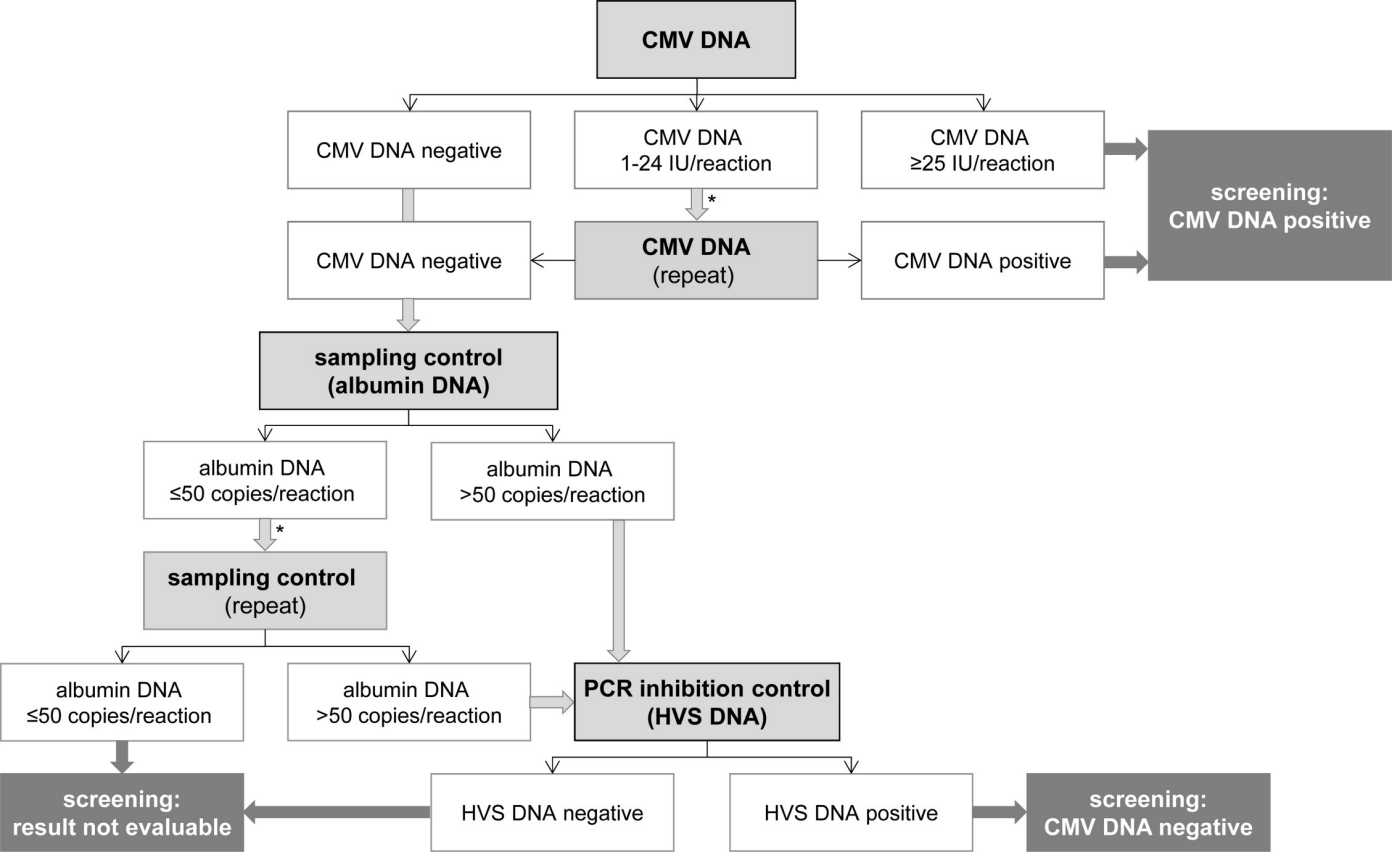
Flow chart of the cCMV screening algorithm. Buccal swabs were tested for the presence of CMV DNA. Samples that contained ≥25 IU CMV DNA/PCR reaction were considered as CMV DNA positive. Samples that were positive but contained <25 IU CMV DNA/PCR reaction were re-tested. For saliva samples that were tested negative in the initial or repeated CMV DNA, PCR internal controls (albumin, Herpesvirus saimiri (HVS)) were evaluated to exclude false negative results. *sample re-extraction and PCR black thin arrow: test result; light gray arrow: subsequent investigation; dark gray arrow: clinical report.

Buccal swab samples were taken from newborns within the first three days of life. CMV DNA, albumin DNA (cellular control) and HVS DNA (PCR extraction and inhibition control) were detected simultaneously with a multiplex real-time PCR developed and validated in our laboratory. As illustrated in Fig 1, a laboratory test algorithm was generated for the interpretation of the PCR results. Saliva samples with more than ≥25 IU CMV DNA/PCR reaction (n = 24) were classified as CMV DNA screening positive. For samples with 1 to 24 IU CMV DNA/PCR reaction (n = 34) DNA was isolated from the original eNAT^™^ sample by a different extraction method and re-analyzed by PCR. 14 of the 34 retested samples were confirmed to contain CMV DNA by the second independent analysis and were also classified as CMV DNA screening positive. The remaining 20 of the 34 retested samples were assessed as CMV DNA negative. To avoid false negative results due to inefficient sampling, the albumin DNA content was determined. CMV DNA negative screening samples with less than or equal to 50 copies of albumin per PCR reaction (n = 7) were extracted with EZ1 Virus Mini Kit v2.0 and re-tested again. In three cases samples remained below 50 albumin copies per PCR reaction and were therefore classified as not evaluable. Finally, detection of HVS in appropriate amounts was assessed in all CMV DNA negative samples whose albumin DNA levels exceeded 50 copies per PCR reaction to exclude false negative findings resulting from PCR inhibition. HVS DNA was detected in all these buccal swabs at concentrations within the expected range.

**Fig 2:**
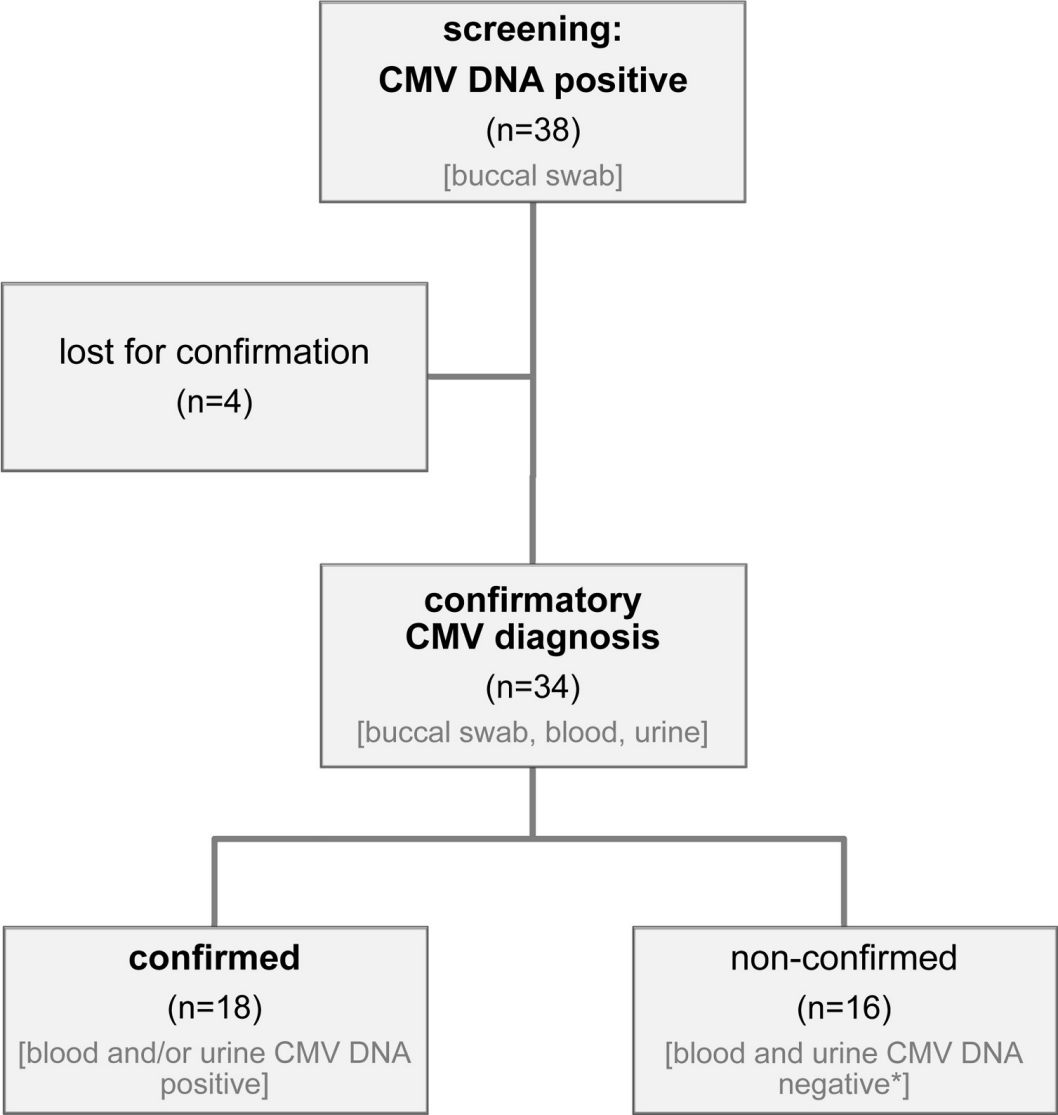
Flow chart of confirmatory CMV diagnostics. 34 out of 38 patients whose saliva samples (buccal swabs) were tested positive in CMV DNA screening could be included in confirmatory diagnostics. Confirmation of cCMV infection was defined as CMV DNA detection in blood and/or urine. *including 3 swab samples that were again tested positive for CMV DNA.

Introducing the test algorithm illustrated in Fig 1, 38 out of the tested buccal swabs were found to be CMV DNA positive (Fig 2). 34 of these children could be enrolled for confirmatory diagnostics including CMV DNA determination in urine and blood. In 13 of these 34 cases CMV DNA was undetectable in a second buccal swab, urine and blood taken during the first weeks of life. All these children showed only a low viral load of CMV DNA in the screening assay (2 – 3x10^2^ IU CMV DNA/PCR reaction). In three of the 34 included newborns, CMV DNA could only be detected in small quantities in the second buccal swab (17–3.2x10^2^ IU CMV DNA/PCR reaction), but not in urine or blood samples. Because contamination of the swab samples or local transient infection of the oral cavity without systemic infection could not be excluded and since the clinical relevance of cCMV infections in the absence of viremia and viruria can be questioned, these three samples were also classified as non-confirmed (total n = 16, results of multiplex PCR of saliva samples see S1 Table). The remaining 18 cases had detectable levels of CMV DNA in the urine and/or the blood (in addition to the second buccal swab) and were therefore classified as confirmed cCMV infections (S1 Table). In the screening test, the median viral load in buccal swabs of confirmed samples (1.2x10^5^ IU CMV DNA/PCR reaction) was significantly higher than the one of non-confirmed samples (9 IU CMV DNA/PCR reaction; Mann-Whitney-U-Test: p<0.0001).

### CMV DNA load in samples of cCMV infected newborns

**Fig 3:**
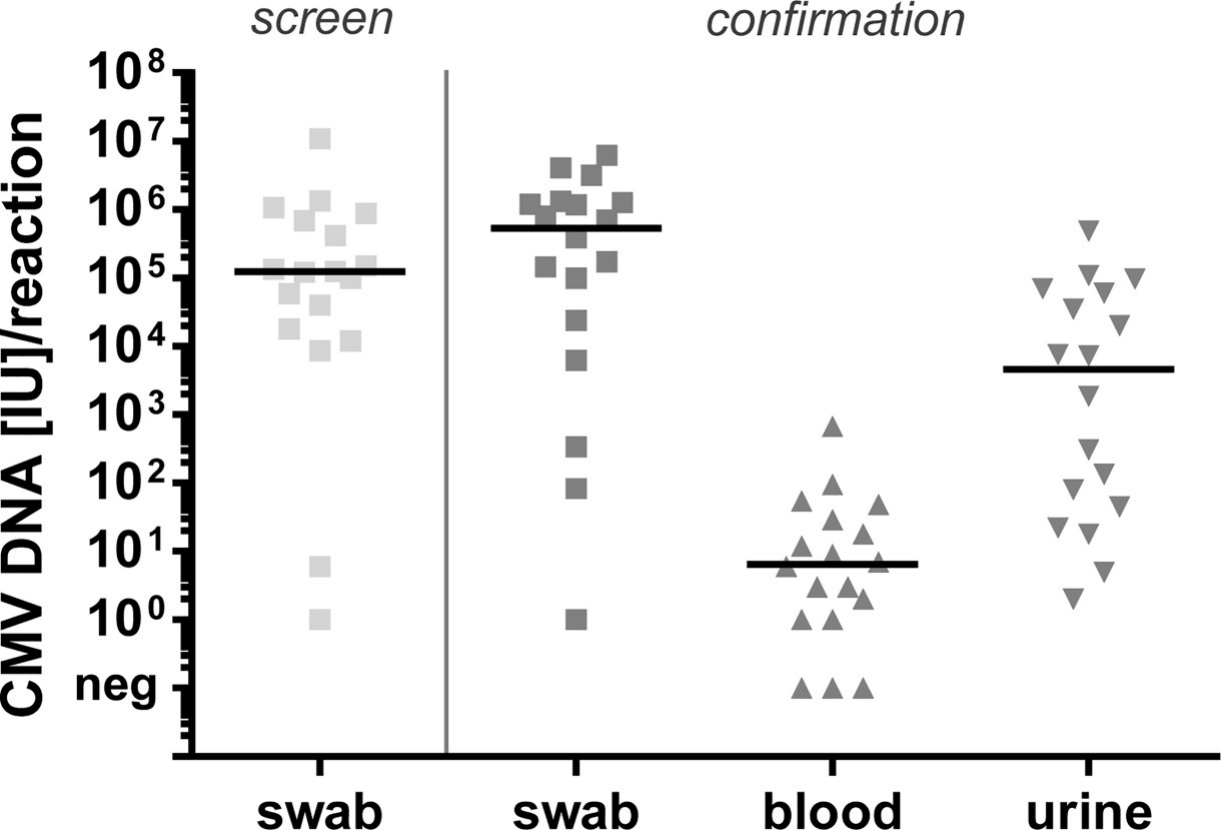
CMV DNA load in samples of study participants with confirmed cCMV infection. Fig 3 illustrates the viral load (IU CMV DNA/PCR reaction) in samples of the 18 newborns with confirmed cCMV infection. CMV DNA loads in buccal swabs at the time of the screening test (left) are compared to the CMV DNA loads in buccal swab, blood and urine at the time of confirmatory diagnostics (right). Medians are represented as black lines.

Samples for screening and confirmatory diagnostics of the 18 neonates with confirmed cCMV infection were analyzed regarding the CMV DNA load in different materials. As shown in Fig 3, the highest amounts of CMV DNA were detected in buccal swabs regardless of the time of sampling (screen *vs*. confirmation). The viral load in swabs is even significantly higher than in urine (Wilcoxon signed rank test: p = 0.0019), which previously served as gold standard for laboratory diagnosis of cCMV infection. In all children CMV DNA was detected in swabs (1–6.3x10^6^ IU/PCR reaction) and urine (2–4.9x10^5^ IU/PCR reaction) at confirmatory diagnostics. In contrast, CMV DNA load in EDTA blood was below 6.7x10^2^ IU/PCR reaction in all samples and CMV DNA could not be detected at all in 3 of 18 (17%) blood samples. The low viral load levels in blood compared to buccal swabs are not due to different elution volumes during DNA extraction, because the substantial difference remains after correction for the elution volume expressing all results as IU/ml (S3 Table). Therefore, EDTA blood also seems less suitable for confirmatory laboratory diagnostics of cCMV infection in newborns than urine.

**Fig 4:**
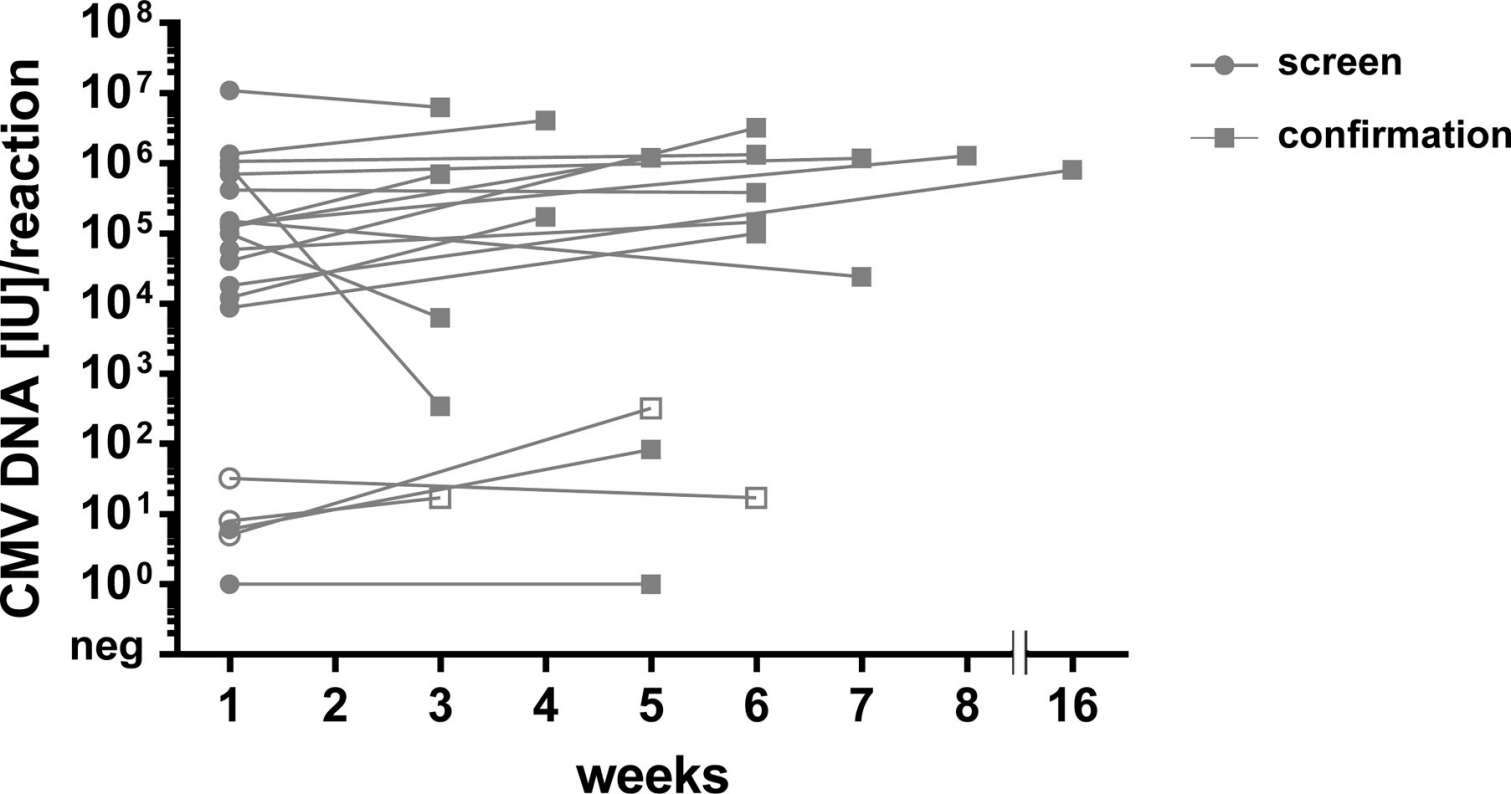
Course of CMV DNA load in saliva samples. The course of CMV DNA loads (IU/PCR reaction) in buccal swabs from screening and confirmatory testing is shown for each individual patient. Included were all patients with confirmed cCMV infection (n = 18, filled icons) as well as the three patients who were found to be CMV DNA positive in the buccal swab but not in blood and urine at time of confirmation (open symbols). Samples for CMV screening were taken within 3 days after birth (week 1, represented as gray circle). Sampling for confirmatory diagnostics was performed in five cases within in the recommended time frame of three weeks after birth and in 15 cases within three and eight weeks after birth (figured as gray square). In one case confirmatory diagnostics could only be done after 16 weeks.

The course of CMV DNA load in buccal swabs at the time of screening during the first three days of life and on confirmation for each individual patient is illustrated in Fig 4. The CMV DNA load did not differ significantly between the two time points (Wilcoxon signed rank test for samples of confirmed cCMV infection: p = 0.1089).

### Testing pools of saliva samples

Based on the high CMV DNA loads in the confirmed cCMV infections, pooling of saliva samples of newborns for high-throughput laboratory testing may be possible to improve cost effectiveness. To determine the sensitivity of pool testing 20 μl of serial dilutions of two screening positive saliva samples in eNAT^™^ medium were mixed with 180 μl of an eNAT^™^ pool of 20 CMV screening negative saliva samples prior to DNA extraction and PCR analyses. The pools tested CMV DNA positive if the viral load of the initial serial dilutions was >50 IU CMV DNA/PCR reaction in accordance with the lower detection limit of the CMV PCR (S4 Table). Retesting of 17 confirmed CMV positive saliva samples after a 1:10 dilution in an eNAT^™^ pool of 20 CMV screening negative saliva samples resulted in positive CMV DNA tests with the exception of two positive saliva samples with very low viral load levels (1 and 6 IU CMV/DNA per PCR reaction) during the initial screening assay (S5 Table). These results confirm the suitability of pool testing for universal screening. Furthermore, retesting of screening samples (eNAT^™^ medium containing DNA from buccal swabs) after being frozen at -20°C for up to three years after sampling showed only minor deviations from the CMV DNA load at the time of initial PCR screening (median deviation retesting/screening: 18.6%, S5 Table) demonstrating the excellent features of eNAT^™^ medium for DNA stabilization even over a long period of time.

### Correlation of CMV DNA loads in buccal swab and urine samples

**Fig 5:**
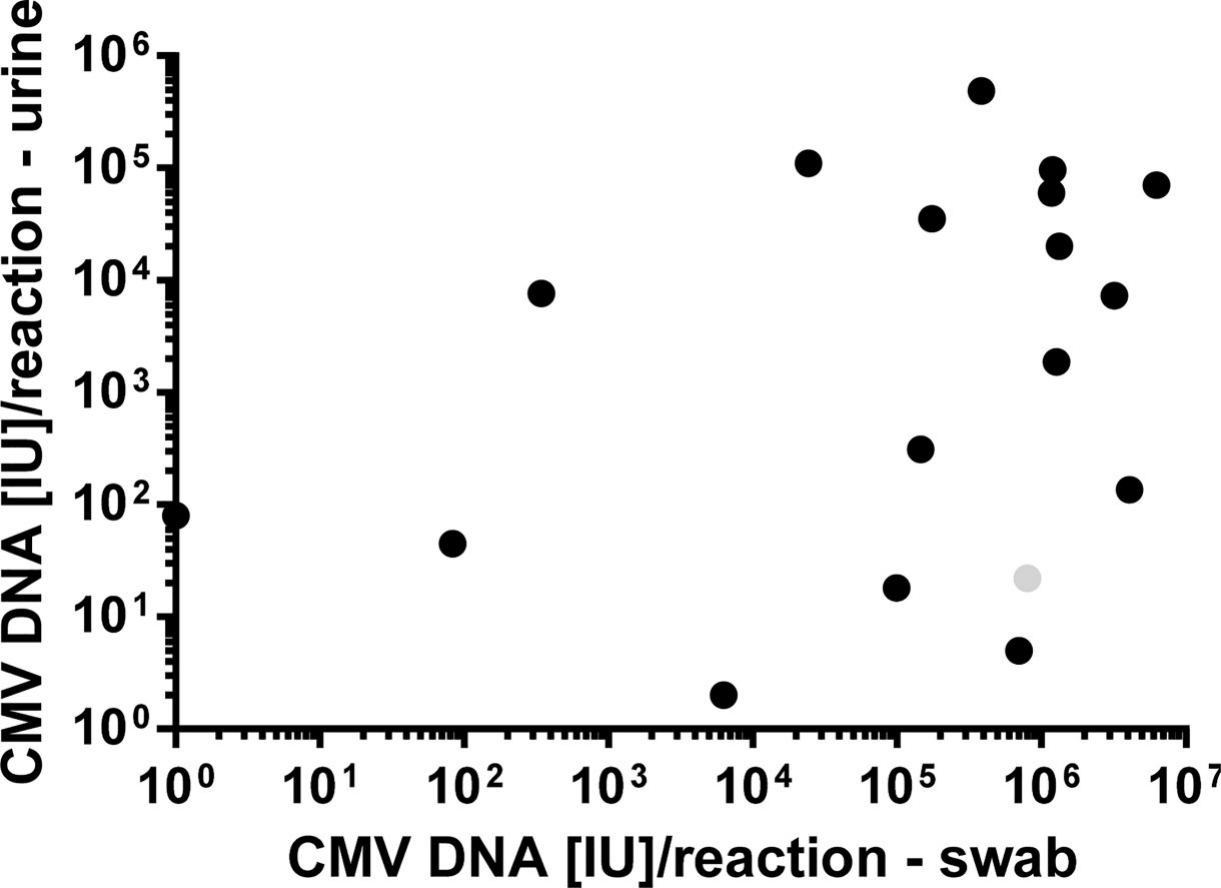
Correlation of CMV DNA loads in urine and saliva swabs. Correlation of CMV DNA loads in buccal swab and urine, respectively, of the 18 newborns with cCMV infection at the time of confirmatory diagnostics is pictured. Statistical analysis showed no correlation (Spearman´s rank correlation coefficient: r = 0.3065, p = 0.2161). The measured pair of values from the patient whose confirmatory diagnostics could be done only after 16 weeks of life is illustrated as gray circle.

As shown in Fig 3 the viral load in buccal swabs is significantly higher than in urine at the time of confirmatory diagnostics. However, the CMV DNA loads in both materials did not correlate (Fig 5, Spearman´s rank correlation coefficient: r = 0.3065, p = 0.2161). This could be due to the fact that urine was stored at -20°C for better handling, since storage of fresh urine at 4°C or lower temperatures is described to result in significant DNA degradation of varying degrees ([15, 16]; own data S6 Table). However, in six out of 18 patients buccal swabs and urine were sent immediately after sampling (without freezing). In five of these six children the CMV DNA load was also higher in the buccal swabs (9.9x10^4^–3.2x10^6^ IU/PCR reaction) than in urine (5–9.7x10^4^ IU/PCR reaction) and viral loads in the two materials of these six patients did not correlate either.

### Distribution of CMV DNA load of all newborns tested positive in cCMV screening

**Fig 6:**
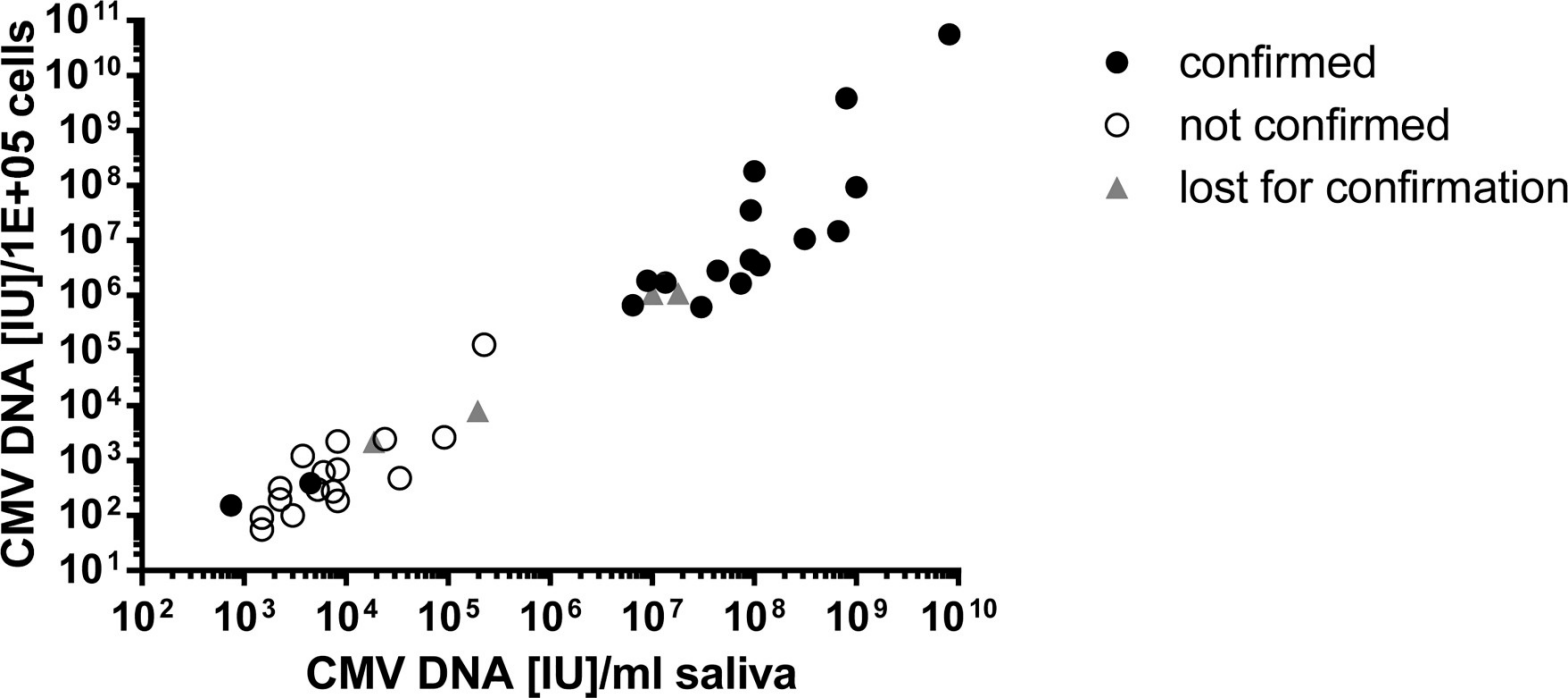
Distribution of CMV DNA load in screening samples of participants with confirmed/non-confirmed cCMV infection. The correlation of CMV DNA loads in buccal swabs expressed as IU CMV DNA/10^5^ cell equivalents and IU CMV DNA/ml saliva of newborns tested positive in CMV PCR at the time of screening is shown. Patients were classified as “confirmed”, “not confirmed” and “lost for confirmation”, respectively, with regard to the result of confirmatory diagnostics.

The quantity of CMV DNA was reported as IU/PCR reaction (Figs 3–5). To control for swabbing efficiency, the number of cells contained in the buccal swabs (detected by albumin DNA content) could be calculated and CMV DNA load can be expressed as IU/10^5^ cell equivalents. Furthermore, viral load can be expressed as IU CMV DNA/ml saliva (S3 Table). As expected, CMV DNA loads expressed as IU of CMV DNA/ml saliva and IU of CMV DNA per 10^5^ cell equivalents showed a close correlation (Spearman´s rank correlation coefficient (all samples): r = 0.9596, p<0.0001, Fig 6). The graph clearly separates confirmed CMV DNA positive samples from those that could not be confirmed. All the 16 non-confirmed samples had a viral load below 1.3x10^5^ IU CMV DNA/10^5^ cell equivalents, 2.3x10^5^ IU/ml, and 3x10^2^ IU CMV DNA/PCR reaction, respectively. In contrast, 16 out of the 18 confirmed samples had viral loads above 6.2x10^5^ IU CMV DNA/10^5^ cell equivalents, 6.4x10^6^ IU CMV DNA/ml saliva, or 8.6x10^3^ IU CMV DNA/PCR reaction, respectively. The two participants with confirmed screening samples falling in the low viral load range also had low viral load levels at confirmatory testing of urine, blood, and saliva at 5 weeks of age (Table 2). For assessment of these patients as possibly CMV DNA positive at time of screening despite values ​​close to the detection limit of the CMV DNA PCR, the original eNAT^™^ samples were retested using a different DNA extraction method and re-analyzing the sample by PCR, as described in Fig 1. It will be important to follow up these patients in order to be able to define screening positive children with high or low-risk of late-onset CMV disease.

**Table 2:** Characteristics of cCMV patients with very low levels of CMV DNA in screening.

patient ID	sex	screening		confirmation				clinical symptoms at birth
		sampling (days after birth)	CMV DNA (buccal swab)	sampling (weeks after birth)	CMV DNA (buccal swab)	CMV DNA (EDTA blood)	CMV DNA (urine)	
#1	female	2	1 IU/PCR reaction = 154 IU/10^5^ cells = 750 IU/ml	5	1 IU/PCR reaction = 24 IU/10^5^ cells = 750 IU/ml	95 IU/PCR reaction = 4,750 IU/ml	80 IU/PCR reaction = 4,000 IU/ml	asymptomatic
#2	male	1	6 IU/PCR reaction = 387 IU/10^5^ cells = 4,500 IU/ml	5	83 IU/PCR reaction = 7,217 IU/10^5^ cells = 62,250 IU/ml	9 IU/PCR reaction = 450 IU/ml	45 IU/PCR reaction = 2,250 IU/ml	asymptomatic

## Discussion

Congenital CMV infection is a relevant cause of neurological and sensory disabilities in childhood. The majority of infants with cCMV infection are asymptomatic at birth and typically not diagnosed. Approximately 10 to 15% of them develop late-onset sequelae like sensorineural hearing loss stressing the need for a postnatal CMV screening of newborns [17, 18] to allow an early diagnosis, follow up and intervention. Since screening approaches based on the occurrence of characteristic clinical manifestations at birth harbor the risk of missing asymptomatic cases, recent studies propose the implementation of a universal screening for cCMV infections [9] and show its cost-effectiveness under a wide range of assumptions [2, 18–20]. However, since the majority of cCMV infected children that are asymptomatic at birth never develop clinical symptoms, there is also a need to limit the number of parents that are unnecessarily worried by their children getting diagnosed as having a cCMV infection. We therefore explored a well-controlled quantitative PCR and developed a test algorithm for the screening and confirmation of cCMV infection of newborns which are appropriate for the utilization in a universal screening approach with centralized laboratory testing. Our results confirm previous reports showing that buccal swabs are suitable for the diagnosis of cCMV infection [7, 8]. Our quantitative analysis of CMV DNA loads in saliva screening samples leads to similar results as those obtained in the study of Leruez-Ville et al. [21] following a comparable approach. The median viral loads of confirmed samples are 7.6 log_10_ DNA copies/ml in their and 5.1 log_10_ IU of CMV DNA/ml in our study (the calibration by WHO standard revealed a conversion factor of 1.0, i.e. IU/ml = copies/ml in our CMV PCR), while the median viral loads in non-confirmed samples are 2.2 and 1.0 log_10_, respectively. The positive predictive value reported by Leruez-Ville et al. is 58.6% compared to 52.9% in our study, based on a positive urine and/or blood sample during confirmatory analysis. If we also consider cases having only positive saliva samples in the confirmatory analysis as true positives, as done by Leruez-Ville et al., the positive predictive value in our study rises to 61.8%. In either case, the modest positive predictive value of the screening approach requires confirmatory CMV diagnosis of screening positive neonates [21].

Both saliva and urine specimens are considered appropriate for neonatal PCR-based CMV screening [5, 22, 23]. We and others observed significantly higher viral load levels in the buccal swabs samples than in blood and urine samples (Fig 3; [24]). The higher content of CMV DNA and much easier collection and handling suggest that buccal swabs are preferable to urine and EDTA blood samples in neonatal CMV screening programs. In particular, the use of saliva samples improved the inclusion rate in screening programs since many newborns had to be excluded due to unsuccessful sampling of urine [6]. Viral load levels in EDTA blood were more than 10^4^-fold lower than in buccal swabs (Fig 3, S3 Table), consistent with the reduced sensitivity reported for assays based on dried blood spots [22, 25–28]. A recent study on symptomatic cCMV infections even revealed that CMV DNA levels were undetectable in 11% of whole blood samples of clinically symptomatic patients further questioning congenital CMV screening approaches based on blood samples [29]. Although median viral load levels did not differ significantly in the swab samples obtained during screening and confirmation, one caveat of our study is that the comparison to viral load levels in blood and urine was restricted to the confirmatory analysis. In addition, the predictive values of positive swab, urine and blood samples for development of late onset disease remain to be compared.

CMV DNA detection in saliva samples by PCR revealed positive screening results that could not be confirmed in follow-up samples (e.g. [7, 8, 21, 26]). Contamination of neonatal saliva samples by CMV DNA present in genital secretions in the birth canal during delivery [10] or in the milk remaining from the last breast feeding [11, 30] are possible reasons for that. Ross et al. concluded that breastfeeding in the first weeks of life contributes to low but acceptable rates (0.03–0.14%) of false positive saliva PCR results [31]. However, amounts of CMV DNA due to contamination with genital secretions or mother´s milk after breastfeeding should be smaller than CMV DNA levels resulting from cCMV infection. Consistently, participants with false positive saliva screening samples had significantly lower viral load levels than participants with confirmed congenital CMV infection (see above, [1, 21, 31]. The percentage of false positive screening results also differed largely between the studies varying from 7.5% to 41% of samples positive in the initial screen. This may be explained by differing analytical sensitivities of the particular screening approaches or differences in the sampling procedures such as immediate sampling in the delivery room *versus* sampling within three days after birth.

However, the non-confirmed positive screening results could also be explained by local transient infections of the oral cavity of the newborns after exposure to CMV containing genital fluids or breast milk. A recent study by Mayer et al. [12] investigating oral virus shedding in 30 Ugandan infants found, that CMV DNA could transiently be detected in oral swabs of newborns who had not acquired systemic primary infection. The self-limited episodes of oral virus shedding were brief (mostly <13d), but in some infants recurrent transient infections were determined. In accordance with the observed lower viral load in buccal swabs of infants whose cCMV infection could not be confirmed in our and others studies, the authors described that transient infections were remarkable for comparatively low CMV DNA loads (median 3.5 (2.3–5.5) log_10_ copies/swab *versus* 7.5 (4.3–8.9) log_10_ copies/swab in infants with primary infection). A very small number of initially infected epithelial cells in the oral cavity and low viral infectivity are suspected as reasons for such transient CMV infection episodes [12]. Three of our cases with positive saliva screening result were also weakly positive for the confirmatory saliva samples (Fig 4), but CMV DNA negative in the confirmatory urine and blood samples. Since contamination of saliva samples by breast milk is particularly unlikely at the confirmatory sampling due to a minimal two hour time interval between sampling and the last breast feeding these repeatedly CMV DNA positive samples provide further support for the transient oral infection hypothesis. These results also suggest that the confirmation of congenital CMV infection in children with low viral load levels in saliva samples during a universal screening may be better based on urine samples than on a second saliva sample. This is consistent with a previous recommendation for confirmatory diagnosis of cCMV infection in children born to mothers with diagnosed primary CMV infection during pregnancy [32].

One limitation of our study is that the sample collection for confirmatory diagnostics was outside the recommended time frame of three weeks in 14 of 18 newborns, who were classified as cCMV infected based on the results of the confirmatory testing. Although we tried to perform sampling for confirmatory diagnostics as soon as possible after obtaining a positive screening result, unfortunately not all children were available for confirmatory sampling within the first three weeks of life. Therefore, we cannot formally exclude that a minority of the 14 cases for which the cCMV infection was confirmed after the first three weeks of life are false-positively screened cases with a postnatal CMV infection. The high viral load observed during screening in 12 of these 14 cases argues against this. In addition, available CMV seroprevalence data for one to two year old children indicate that the upper limit for the frequency of such postnatal CMV infections is about 8% for one-year-olds and 22% for two year olds [33, 34]. Therefore, the probability that CMV infection in more than one of the 14 cases, whose confirmatory diagnostics was carried out later than three but less than 17 weeks of age, was actually due to postnatal infection is very low.

In summary, combining the commercially available eNAT^™^ medium with excellent virus recovery rates and a quantitative CMV multiplex real-time PCR results in a distinguished test system for a universal cCMV screening strategy that should be compatible with a centralized testing strategy of pooled saliva samples. Clinical follow-up of cCMV infections with low viral load levels in saliva screening samples is necessary to define a viral load cut-off below which the risk for late-onset CMV diseases is negligible.

## Supporting information

S1 Table A. Summary of the multiplex PCR data of buccal swabs of newborns with confirmed cCMV infection. B. Summary of the multiplex PCR data of buccal swabs of newborns, whose CMV DNA positive screening result was not confirmed.
(DOCX)

S2 Table Influence of storage conditions on CMV DNA recovery. The flocked swabs of the eNAT^™^ kit (Copan Italia, Bresca, Italy; order number: 608CS01R), were immersed in a virus suspension with rotating movements for 4–6 seconds and subsequently transferred into 1 ml of eNAT^™^ medium or 1 ml virus transport medium (Sigma-Virocult, Medical Wire & Equipment, Corsham, Wiltshore, UK) as described by Kohmer et al. [13]. After a storage time of eight days at room temperature reflecting a reasonable time interval between sampling and laboratory testing in centralized screening approaches, in a part of the samples DNA was extracted from 200μl and eluted in 60μl as described in the Material and Methods section prior CMV PCR. In the other part of the samples PCR was performed without prior DNA extraction.
(DOCX)

S3 Table CMV DNA load in buccal swab, EDTA blood and urine, respectively. (DOCX)

S4 Table Serial dilutions of CMV DNA positive saliva samples were mixed with a pool of CMV DNA negative saliva samples. Serial dilutions of two screening positive saliva samples in eNAT^™^ medium were prepared and CMV DNA was quantified. Pool testing was performed using 20 μl of original sample or dilution mixed with 180 μl of an eNAT^™^ pool containing 20 CMV negative saliva samples.
(DOCX)

S5 Table Pool testing of CMV screening positive saliva samples. Retesting of 17 confirmed CMV positive saliva samples after a 1:10 dilution in an eNAT^™^ pool of 20 CMV screening negative saliva samples.
(DOCX)

S6 Table CMV DNA levels in fresh *versus* frozen (-20°C) urine. CMV DNA levels in urine of 12 patients were quantified by real time PCR immediately after receipt of samples and after storage at -20°C for the indicated time.
(DOCX)
