# Supplementary material for: Characterization of a universal screening approach for congenital CMV infection based on a highly-sensitive, quantitative, multiplex real-time PCR assay
Source: PLoS One. 2020 Jan 9;15(1):e0227143. doi: 10.1371/journal.pone.0227143 (PMC6952102; doi:10.1371/journal.pone.0227143)
Supplement: S1 Table — A. Summary of the multiplex PCR data of buccal swabs of newborns with confirmed cCMV infection. B. Summary of the multiplex PCR data of buccal swabs of newborns, whose CMV DNA positive screening result was not confirmed. (DOCX) [file pone.0227143.s001.docx]

**S1a Table. Summary of the multiplex PCR data of buccal swabs of newborns with confirmed cCMV infection.**

| patient ID | **screening** | | | **confirmation** | | |
| --- | --- | --- | --- | --- | --- | --- |
|  | CMV DNA  [IU/PCR reaction] | albumin DNA  [copies/PCR reaction] | HVS DNA  [Ct] | CMV DNA  [IU/PCR reaction] | albumin DNA  [copies/PCR reaction] | HVS DNA  [Ct] ^a)^ |
| #1 | 1 | 1.3x10^3^ | 29.1 | 1 | 8.5x10^3^ | 31.1 |
| #2 | 6 | 3.1x10^3^ | 29.2 | 8.3x10^1^ | 2.3x10^3^ | 28.4 |
| #3 | 8.6x10^3^ | 2.6x10^3^ | 28.4 | 9.9x10^4^ | 5.2x10^2^ | 29.4 |
| #4 | 1.2x10^4^ | 1.3x10^3^ | 29.0 | 1.7x10^5^ | 4.3x10^2^ | 30.2 |
| #5 | 1.8x10^4^ | 2.1x10^3^ | 27.7 | 8.0x10^5^ | 7.4x10^2^ | 31.8 |
| #6 | 4.0x10^4^ | 1.3x10^4^ | 27.1 | 3.2x10^6^ | negative ^b)^ | 38.8 ^c)^ |
| #7 | 5.9x10^4^ | 4.2x10^3^ | 28.2 | 1.5x10^5^ | 5.9x10^2^ | 28.4 |
| #8 ^d)^ | 9.9x10^4^ | 1.2x10^4^ | 28.7 | 6.3x10^3^ | 2.3x10^3^ | 28.1 |
| #9 ^d)^ | 1.2x10^5^ | 5.5x10^3^ | 32.2 | 7.0x10^5^ | 2.2x10^1 b)^ | 33.5 |
| #10 | 1.2x10^5^ | 7.0x10^2^ | 28.4 | 1.2x10^6^ | negative ^b)^ | 31.7 |
| #11 | 1.3x10^5^ | 1.5x10^2^ | 29.0 | 1.3x10^6^ | 2.1x10^1 b)^ | 32.7 |
| #12 | 1.5x10^5^ | 8.5x10^3^ | 30.6 | 2.4x10^4^ | 3.7x10^2^ | 28.8 |
| #13 | 4.2x10^5^ | 7.9x10^3^ | 30.4 | 3.8x10^5^ | 2.3x10^3^ | 29.8 |
| #14 | 6.9x10^5^ | negative ^b)^ | 29.2 | 1.2x10^6^ | negative ^b)^ | 31.1 |
| #15 ^d)^ | 8.9x10^5^ | 1.2x10^4^ | 32.2 | 3.4x10^2^ | 2.9x10^3^ | 29.1 |
| #16 | 1.1x10^6^ | 5.5x10^1 b)^ | 38.7 ^c)^ | 1.3x10^6^ | 6 ^b)^ | 39.1 ^c)^ |
| #17 | 1.3x10^6^ | 2.9x10^3^ | 30.1 | 4.1x10^6^ | 2 ^b)^ | 33.6 |
| #18 ^d)^ | 1.1x10^7^ | 3.9x10^1 b)^ | 37.8 ^c)^ | 6.3x10^6^ | negative ^b)^ | 39.3 ^c)^ |

^a)^  target value: Ct < 34

^b)^  low concentrations of albumin DNA as result of very high concentration of CMV DNA in the sample (suppression/inhibition of further targets in the multiplex real-time PCR assay due to

competition regarding nucleotides, enzyme etc.)

^c)^  higher Ct value of HVS DNA as result of very high concentration of CMV DNA in the sample (suppression/inhibition of further targets in the multiplex real-time PCR assay due to competition

regarding nucleotides, enzyme etc.)

^d)^  confirmatory diagnostics was performed within the recommended time frame of three weeks after birth, in the other cases sample collection for confirmation was carried out within 8 weeks

(exception patient #5: 16 weeks) after birth

**S1b Table. Summary of the multiplex PCR data of buccal swabs of newborns, whose CMV DNA positive screening result was not confirmed.**

| patient ID | **screening** | | | **confirmation** | | |
| --- | --- | --- | --- | --- | --- | --- |
|  | CMV DNA  [IU/PCR reaction] | albumin DNA  [copies/PCR reaction] | HVS DNA  [Ct] | CMV DNA  [IU/PCR reaction] | albumin DNA  [copies/PCR reaction] | HVS DNA  [Ct] ^a)^ |
| #19 | 2 | 4.4x10^3^ | 28.8 | negative | 8.9x10^3^ | 29.0 |
| #20 | 2 | 7.2x10^3^ | 29.1 | negative | 5.3x10^3^ | 28.4 |
| #21 ^c)^ | 3 | 3.1x10^3^ | 28.4 | negative | 5.1x10^3^ | 28.7 |
| #22 | 3 | 1.9x10^3^ | 27.1 | negative | 8.2x10^3^ | 29.2 |
| #23 | 4 | 8.0x10^3^ | 29.3 | negative | 3.6x10^3^ | 28.5 |
| #24 | 7 | 4.7x10^3^ | 27.8 | negative | 8.3x10^3^ | 28.4 |
| #25 ^c)^ | 1.0x10^1^ | 7.3x10^3^ | 28.8 | negative | 3.6x10^3^ | 29.1 |
| #26 | 1.1x10^1^ | 9.9x10^2^ | 29.1 | negative | 3.5x10^3^ | 28.4 |
| #27 | 1.1x10^1^ | 1.2x10^4^ | 29.9 | negative | 1.4x10^3^ | 28.4 |
| #28 | 1.1x10^1^ | 3.2x10^3^ | 28.4 | negative | 1.2x10^3^ | 28.7 |
| #29 ^c)^ | 4.5x10^1^ | 1.9x10^4^ | 29.0 | negative | 1.3x10^4^ | 27.3 |
| #30 | 1.2x10^2^ | 9.2x10^3^ | 28.7 | negative | 8.0x10^3^ | 29.7 |
| #31 | 3.0x10^2^ | 4.7x10^2^ | 28.6 | negative | 6.2x10^3^ | 28.8 |
| #32 ^b)^ | 5 | 8.3x10^2^ | 28.7 | 3.2x10^2^ | 2.3x10^3^ | 29.2 |
| #33 ^b), c)^ | 8 | 2.6x10^3^ | 28.9 | 1.7x10^1^ | 1.6x10^3^ | 27.9 |
| #34 ^b)^ | 3.2x10^1^ | 2.6x10^3^ | 28.2 | 1.7x10^1^ | 9.6x10^3^ | 29.8 |

^a)^  target value: Ct < 34

^b)^  at confirmation CMV DNA was detected in low concentration in the buccal swab, but not in urine or blood samples. Therefore, these newborns were classified as non-confirmed

^c)^  confirmatory diagnostics was performed within the recommended time frame of three weeks after birth, in the other cases sample collection for confirmation was carried out within 8 weeks

after birth
