# Supplementary material for: Characterization of a universal screening approach for congenital CMV infection based on a highly-sensitive, quantitative, multiplex real-time PCR assay
Source: PLoS One. 2020 Jan 9;15(1):e0227143. doi: 10.1371/journal.pone.0227143 (PMC6952102; doi:10.1371/journal.pone.0227143)
Supplement: S2 Table — The flocked swabs of the eNAT™ kit (Copan Italia, Bresca, Italy; order number: 608CS01R), were immersed in a virus suspension with rotating movements for 4–6 seconds and subsequently transferred into 1 ml of eNAT™ medium or 1 ml virus transport medium (Sigma-Virocult, Medical Wire & Equipment, Corsham, Wiltshore, UK) as described by Kohmer et al. [13]. After a storage time of eight days at room temperature reflecting a reasonable time interval between sampling and laboratory testing in centralized screening approaches, in a part of the samples DNA was extracted from 200μl and eluted in 60μl as described in the Material and Methods section prior CMV PCR. In the other part of the samples PCR was performed without prior DNA extraction. (DOCX) [file pone.0227143.s002.docx]

**S2 Table. Influence of storage conditions on CMV DNA recovery.**

| Experimental setting | CMV DNA IU/PCR reaction [mean (± SD)] | CMV DNA IU/ml  [mean (± SD)] |
| --- | --- | --- |
| eNAT™ with DNA extraction | 395 (± 157) | 11,850 (± 4,722) |
| Virocult with DNA extraction | 87 (± 28) | 2,600 (± 840) |
| Virocult without DNA extraction | 60 (± 45) | 1,810 (± 1,349) |

The flocked swabs of the eNAT™ kit (Copan Italia, Bresca, Italy; order number: 608CS01R), were immersed in a virus suspension with rotating movements for 4-6 seconds and subsequently transferred into 1 ml of eNAT™ medium or 1 ml virus transport medium (Sigma-Virocult, Medical Wire & Equipment, Corsham, Wiltshore, UK) as described by Kohmer et al. [Kohmer et al., J Clin Virol, 2019, 115: 32-36]. After a storage time of eight days at room temperature reflecting a reasonable time interval between sampling and laboratory testing in centralized screening approaches, in a part of the samples DNA was extracted from 200µl and eluted in 60µl as described in the Material and Methods section prior CMV PCR. In the other part of the samples PCR was performed without prior DNA extraction.
