# Supplementary material for: Characterization of a universal screening approach for congenital CMV infection based on a highly-sensitive, quantitative, multiplex real-time PCR assay
Source: PLoS One. 2020 Jan 9;15(1):e0227143. doi: 10.1371/journal.pone.0227143 (PMC6952102; doi:10.1371/journal.pone.0227143)
Supplement: S3 Table — (DOCX) [file pone.0227143.s003.docx]

**S3 Table. CMV DNA load in buccal swab, EDTA blood and urine, respectively.**

| patient ID | **screening** | | **confirmation** | | | | | |
| --- | --- | --- | --- | --- | --- | --- | --- | --- |
|  | buccal swab | | buccal swab | | blood | | urine | |
|  | CMV DNA  [IU/PCR reaction] | CMV DNA  [IU/ml] | CMV DNA  [IU/PCR reaction] | CMV DNA  [IU/ml] | CMV DNA  [IU/PCR reaction] | CMV DNA  [IU/ml] | CMV DNA  [IU/PCR reaction] | CMV DNA  [IU/ml] |
| #1 | 1 | 7.5x10^2^ | 1 | 7.5x10^2^ | 9.5x10^1^ | 4.8x10^3^ | 8.0x10^1^ | 4.0x10^3^ |
| #2 | 6 | 4.5x10^3^ | 8.3x10^1^ | 6.2x10^4^ | 9 | 4.5x10^2^ | 4.5x10^1^ | 2.3x10^3^ |
| #3 | 8.6x10^3^ | 6.5x10^6^ | 9.9x10^4^ | 7.4x10^7^ | 1 | 5.0x10^1^ | 1.8x10^1^ | 9.0x10^2^ |
| #4 | 1.2x10^4^ | 9.0x10^6^ | 1.7x10^5^ | 1.3x10^8^ | 5.5x10^1^ | 2.8x10^3^ | 3.5x10^4^ | 1.8x10^6^ |
| #5 | 1.8x10^4^ | 1.3x10^7^ | 8.0x10^5^ | 6.0x10^8^ | negative | negative | 2.2x10^1^ | 1.1x10^3^ |
| #6 | 4.0x10^4^ | 3.0x10^7^ | 3.2x10^6^ | 2.4x10^9^ | 3 | 1.5x10^2^ | 7.3x10^3^ | 3.6x10^5^ |
| #7 | 5.9x10^4^ | 4.4x10^7^ | 1.5x10^5^ | 1.1x10^8^ | 1 | 5.0x10^1^ | 3.1x10^2^ | 1.6x10^4^ |
| #8 | 9.9x10^4^ | 7.4x10^7^ | 6.3x10^3^ | 4.7x10^6^ | 1.8x10^1^ | 9.0x10^2^ | 2 | 1.0x10^2^ |
| #9 | 1.2x10^5^ | 9.2x10^7^ | 7.0x10^5^ | 5.2x10^8^ | 1.2x10^1^ | 6.0x10^2^ | 5 | 2.5x10^2^ |
| #10 | 1.2x10^5^ | 9.3x10^7^ | 1.2x10^6^ | 8.9x10^8^ | 7 | 3.5x10^2^ | 9.7x10^4^ | 4.8x10^6^ |
| #11 | 1.3x10^5^ | 1.0x10^8^ | 1.3x10^6^ | 9.5x10^8^ | 2 | 1.0x10^2^ | 1.9x10^3^ | 9.3x10^4^ |
| #12 | 1.5x10^5^ | 1.1x10^8^ | 2.4x10^4^ | 1.8x10^7^ | negative | negative | 1.1x10^5^ | 5.5x10^6^ |
| #13 | 4.2x10^5^ | 3.1x10^8^ | 3.8x10^5^ | 2.9x10^8^ | negative | negative | 4.9x10^5^ | 2.4x10^7^ |
| #14 | 6.9x10^5^ | 5.2x10^8^ | 1.2x10^6^ | 8.8x10^8^ | 3 | 1.5x10^2^ | 6.0x10^4^ | 3.0x10^6^ |
| #15 | 8.9x10^5^ | 6.6x10^8^ | 3.4x10^2^ | 2.6x10^5^ | 6.7x10^2^ | 3.3x10^4^ | 7.6x10^3^ | 3.8x10^5^ |
| #16 | 1.1x10^6^ | 8.0x10^8^ | 1.3x10^6^ | 9.9x10^8^ | 6 | 3.0x10^2^ | 2.0x10^4^ | 1.0x10^6^ |
| #17 | 1.3x10^6^ | 1.0x10^9^ | 4.1x10^6^ | 3.0x10^9^ | 2.9x10^1^ | 1.5x10^3^ | 1.4x10^2^ | 6.8x10^3^ |
| #18 | 1.1x10^7^ | 8.2x10^9^ | 6.3x10^6^ | 4.7x10^9^ | 4.9x10^1^ | 2.5x10^3^ | 7.1x10^4^ | 3.5x10^6^ |
