# Supplementary material for: Characterization of a universal screening approach for congenital CMV infection based on a highly-sensitive, quantitative, multiplex real-time PCR assay
Source: PLoS One. 2020 Jan 9;15(1):e0227143. doi: 10.1371/journal.pone.0227143 (PMC6952102; doi:10.1371/journal.pone.0227143)
Supplement: S4 Table — Serial dilutions of two screening positive saliva samples in eNAT™ medium were prepared and CMV DNA was quantified. Pool testing was performed using 20 μl of original sample or dilution mixed with 180 μl of an eNAT™ pool containing 20 CMV negative saliva samples. (DOCX) [file pone.0227143.s004.docx]

**S4 Table. Serial dilutions of CMV DNA positive saliva samples were mixed with a pool of CMV DNA negative saliva samples.**

| **Patient ID** | **Dilution** | **Original saliva sample**  **CMV DNA  [IU/PCR reaction)** | **Expected value of pool testing^a^  CMV DNA  [IU/PCR reaction)** | **Pool testing^b^ CMV DNA  [IU/PCR reaction)** |
| --- | --- | --- | --- | --- |
| #16 | undiluted | 1.4x10^6^ | 1.4x10^5^ | 5.0x10^4^ |
|  | 1:10 | 1.4x10^5^ | 1.4x10^4^ | 1.1x10^4^ |
|  | 1:100 | 7.0x10^3^ | 7.0x10^2^ | 6.3x10^2^ |
|  | 1:200 | 5.9x10^3^ | 5.9x10^2^ | 3.4x10^2^ |
|  | 1:500 | 3.0x10^3^ | 3.0x10^2^ | 9.0x10^1^ |
|  | 1:1,000 | 1.5x10^3^ | 1.5x10^2^ | 3.0x10^1^ |
|  | 1:2,000 | 8.0x10^2^ | 8.0x10^1^ | 3.0x10^1^ |
|  | 1:5,000 | 2.5x10^2^ | 2.5x10^1^ | 8 |
|  | 1:10,000 | 1.4x10^2^ | 1.4x10^1^ | 8 |
|  | 1:20,000 | 6.0x10^1^ | 6 | 3 |
|  | 1:50,000 | 4.0x10^1^ | 4 | 1 |
|  | 1:100,000 | 1.0x10^1^ | 1 | not detected |
| #17 | undiluted | 9.7x10^5^ | 9.7x10^4^ | 7.6x10^4^ |
|  | 1:10 | 1.5x10^5^ | 1.5x10^4^ | 1.3x10^4^ |
|  | 1:100 | 1.4x10^4^ | 1.4x10^3^ | 1.1x10^3^ |
|  | 1:200 | 6.8x10^3^ | 6.8x10^2^ | 9.7x10^2^ |
|  | 1:500 | 2.8x10^3^ | 2.8x10^2^ | 1.3x10^2^ |
|  | 1:1,000 | 1.2x10^3^ | 1.2x10^2^ | 6.0x10^1^ |
|  | 1:2,000 | 9.2x10^2^ | 9.2x10^1^ | 2.0x10^1^ |
|  | 1:5,000 | 3.1x10^2^ | 3.1x10^1^ | 9 |
|  | 1:10,000 | 2.0x10^2^ | 2.0x10^1^ | 7 |
|  | 1:20,000 | 8.0x10^1^ | 8 | 1 |
|  | 1:50,000 | 4.0x10^1^ | 4 | 3 |
|  | 1:100,000 | 2.0x10^1^ | 2 | not detected |

Serial dilutions of two screening positive saliva samples in eNAT™ medium were prepared and CMV DNA was quantified. Pool testing was performed using 20 µl of original sample or dilution mixed with 180 µl of an eNAT™ pool containing 20 CMV negative saliva samples.

^a^ expected value: result of original saliva sample divided by dilution factor 10

^b^ original saliva sample/dilution mixed with a pool of CMV negative saliva samples (1:10)
