# Supplementary material for: Characterization of a universal screening approach for congenital CMV infection based on a highly-sensitive, quantitative, multiplex real-time PCR assay
Source: PLoS One. 2020 Jan 9;15(1):e0227143. doi: 10.1371/journal.pone.0227143 (PMC6952102; doi:10.1371/journal.pone.0227143)
Supplement: S5 Table — Retesting of 17 confirmed CMV positive saliva samples after a 1:10 dilution in an eNAT™ pool of 20 CMV screening negative saliva samples. (DOCX) [file pone.0227143.s005.docx]

**S5 Table. Pool testing of CMV screening positive saliva samples.**

| **Patient ID** | **Initial screening assay**  **CMV DNA  [IU/PCR reaction]** | **Repeated screening assay^a^**  **CMV DNA  [IU/PCR reaction]** | **Pool testing^b^ CMV DNA  [IU/PCR reaction)**  **x10** | **% deviation of results of repeated from initial screening assay** | **% deviation of results of pool testing from repeated screening assay** |
| --- | --- | --- | --- | --- | --- |
| Pool^c^ | --- | negative | --- | --- | --- |
| #1 | 1 | negative | negative | --- | --- |
| #2 | 6 | 7 | negative | 16.7 | --- |
| #4 | 1.2x10^4^ | 1.4x10^4^ | 1.4x10^4^ | 16.7 | 0.0 |
| #5 | 1.8x10^4^ | 3.2x10^4^ | 2.5x10^4^ | 77.8 | -21.9 |
| #6 | 4.0x10^4^ | 4.8x10^4^ | 5.6x10^4^ | 20.0 | 16.7 |
| #7 | 5.9x10^4^ | 4.9x10^4^ | 5.3x10^4^ | -16.9 | 8.2 |
| #8 | 9.9x10^4^ | 8.7x10^4^ | 9.7x10^4^ | -12.1 | 11.5 |
| #9 | 1.2x10^5^ | 7.3x10^5^ | 6.4x10^5^ | 508.3 | -12.3 |
| #10 | 1.2x10^5^ | 1.3x10^5^ | 1.2x10^5^ | 8.3 | -7.7 |
| #11 | 1.3x10^5^ | 3.9x10^5^ | 4.0x10^5^ | 200.0 | 2.6 |
| #12 | 1.5x10^5^ | 2.2x10^5^ | 1.7x10^5^ | 46.7 | -22.7 |
| #13 | 4.2x10^5^ | 3.9x10^5^ | 2.4x10^5^ | -7.1 | -38.5 |
| #14 | 6.9x10^5^ | 4.6x10^5^ | 5.9x10^5^ | -33.3 | 28.3 |
| #15 | 8.9x10^5^ | 1.2x10^6^ | 8.6x10^5^ | 34.8 | -28.3 |
| #16 | 1.1x10^6^ | 1.4x10^6^ | 8.3x10^5^ | 27.3 | -40.7 |
| #17 | 1.3x10^6^ | 1.3x10^6^ | 8.6x10^5^ | 0.0 | -33.8 |
| #18 | 1.1x10^7^ | 9.1x10^6^ | 7.4x10^6^ | -17.3 | -18.7 |
| Median  of absolute values |  |  |  | 18.6 | 18.7 |

Retesting of 17 confirmed CMV positive saliva samples after a 1:10 dilution in an eNAT™ pool of 20 CMV screening negative saliva samples.

^a^ Repeated screening assay for up to three years after the initial screening assay as baseline value for pool testing.

^b^ original saliva sample diluted 1:10 in a pool of CMV negative saliva samples, PCR result multiplied by dilution factor 10

^c^ pool of CMV negative saliva samples.
