# Supplementary material for: Characterization of a universal screening approach for congenital CMV infection based on a highly-sensitive, quantitative, multiplex real-time PCR assay
Source: PLoS One. 2020 Jan 9;15(1):e0227143. doi: 10.1371/journal.pone.0227143 (PMC6952102; doi:10.1371/journal.pone.0227143)
Supplement: S6 Table — CMV DNA levels in urine of 12 patients were quantified by real time PCR immediately after receipt of samples and after storage at -20°C for the indicated time. (DOCX) [file pone.0227143.s006.docx]

**S6 Table. CMV DNA levels in fresh *versus* frozen (-20°C) urine.**

| **Patient ID^a^** | **Storage time at  -20°C** [days] | **CMV DNA IU/ml** (fresh urine) | **CMV DNA IU/ml** (frozen urine) | **Difference in CMV DNA amount** [ratio fresh to frozen] |
| --- | --- | --- | --- | --- |
| #35 | 15 | 7.6x10^4^ | 1.1x10^4^ | 6.91 |
| #36 | 39 | 1.3x10^4^ | 1.5x10^2^ | 86.67 |
| #37 | 50 | 1.7x10^6^ | 3.5x10^5^ | 4.86 |
| #38 | 67 | 6.0x10^5^ | 1.1x10^5^ | 5.45 |
| #39 | 88 | 4.1x10^4^ | 4.0x10^3^ | 10.25 |
| #40 | 105 | 1.2x10^6^ | negative | --- |
| #41 | 117 | 1.8x10^7^ | 7.8x10^6^ | 2.31 |
| #42 | 117 | 3.3x10^4^ | 1.2x10^4^ | 2.75 |
| #43 | 121 | 5.2x10^4^ | 5,2x10^4^ | 1.00 |
| #44 | 119 | 3.0x10^4^ | 2.2x10^3^ | 13.64 |
| #15 | 96 | 3.8x10^5^ | 1.2x10^4^ | 31.67 |
| #6 | 2 | 3.6x10^5^ | 8.2x10^4^ | 4.39 |

CMV DNA levels in urine of 12 patients were quantified by real time PCR immediately after receipt of samples and after storage at -20°C for the indicated time.

^a^ #35-44: urine of CMV infected patients not participating in the presented study.
